# Effect of aqueous environment on wear resistance of dental glass–ceramics

**DOI:** 10.1186/s12903-022-02183-5

**Published:** 2022-04-26

**Authors:** Zhenzhen Zhang, Qi Wang, Fu Wang, Ding Li, Meng Meng, Yaming Zhang, Shaofeng Zhang

**Affiliations:** 1 State Key Laboratory of Military Stomatology and National Clinical Research Center for Oral Diseases and Shaanxi Key Laboratory of Oral Diseases, Department of Prosthodontics, School of Stomatology, Fourth Military Medical University, Changle Xi Road 145, Xi’an, 710032 Shaanxi China; 2 State Key Laboratory of Military Stomatology and National Clinical Research Center for Oral Diseases and Shaanxi Key Laboratory of Oral Diseases, Department of Pediatric Dentistry, School of Stomatology, Fourth Military Medical University, Xi’an, 710032 Shaanxi China; 3 School of Science, Xi’an University of Posts and Telecommunications, Xi’an, 710121 Shaanxi China

**Keywords:** Wear, Glass–ceramics, Lithium disilicate, Aqueous environment, Microstructure

## Abstract

**Background:**

Wear resistance affects dental ceramics longevity and the functions of the opposing teeth. However, data for the effect of aqueous environment on wear resistance of dental ceramics are lacking. This study evaluated the effect of aqueous environment on wear resistance of typical dental glass–ceramics.

**Methods:**

Disk specimens were prepared from lithium disilicate glass–ceramics (LD) and leucite reinforced glass–ceramics (LEU). The disk specimens paired with steatite antagonists were tested in a pin-on-disk tribometer under both wet and dry conditions with 10 N up to 500,000 wear cycles. The wear analysis of glass–ceramics was performed using a 3D profilometer after 100,000, 300,000 and 500,000 wear cycles. Wear morphologies were analyzed by employing scanning electron microscopy (SEM). The crystalline compositions of specimens stored in a dry environment and subsequently immersed in distilled water for 40 h were separately determined using X-ray diffraction (XRD). The chemical states of the wear surfaces for LD were analyzed by X-ray photoelectron spectroscopy (XPS). The data analysis and multiple pair-wise comparisons of means were performed by using one-way analysis of variance (ANOVA) and Tukey’s post-hoc test.

**Results:**

LEU in a wet environment exhibited less wear volume loss than that in a dry environment (*p* < 0.05). The volume loss of LD in a wet environment was higher than that in a dry environment (*p* < 0.05). The wear volumes of steatite antagonists paired with two glass–ceramics under dry conditions were higher than under wet conditions.

**Conclusions:**

XPS spectra of LD under wet conditions indicated that high wear loss might result from the effect of stress corrosion by water and reaction of water with the ionic-covalent bonds at the crack tip. XPS spectra and SEM images of LD under dry conditions showed a possible formation of tribofilm. Within the limitations of this in vitro study, water was wear-friendly to LEU and all opposing steatites but aggravated wear for LD.

## Background

Glass–ceramics have been widely used to fabricate a wide variety of restorations including inlays, onlays, implants, crowns and fixed partial dentures on account of their biocompatibility and better esthetics. Due to their better esthetics, patients have become increasingly demanding regarding the appearance of their restorations. Although glass–ceramic restorations have shown enhanced esthetics compared with traditional metal-based and zirconia restorations, their wear resistances still need to be improved [1–3].

Tribological properties are important in the material design and fabrication of dental restorations, which not only determine the restorative longevity but also affect the functions of the opposing teeth. Previous studies have shown that wear behaviors of dental ceramics were influenced by many factors, such as microstructure, mechanical properties, and surface treatment [4–7]. For example, leucite-based glass–ceramics with small-sized crystals induced a reduction in tooth wear according to in vitro study [8]. Meanwhile, the hardness of food, pH values of saliva, and masticatory loads remarkably affected the wear performance of ceramic restorations [9–11]. However, to date, limited information is available regarding the effect of an aqueous environment on the wear resistance of dental glass–ceramics. Patients with xerostomia lack of oral saliva. For them, there is a lack of relevant research on how to choose prosthesis materials and whether the lack of oral saliva affects the performance of ceramics.

Glass ceramics consist of a glass matrix and a crystalline phase [12]. The fundamental structure of glass matrix is a three-dimensional network of silica tetrahedron connected by silicon-oxygen bonds (Si–O–Si). Under the external conditions of temperature less than 250 °C and alkaline or acidic environment, silicon dioxide does not dissolve in water, and the primary corrosion mechanism is the transfer of water molecules into glass phase through ion exchange and Si–O bond cleavage. When the surface energy of glass is close to zero, the hardness of glass fluctuates with increasing surface positive charge. In a humid oral environment, the positive charge will be transferred to the surface of glass–ceramics, and sodium ions in glass–ceramics will be transferred to the water environment, thereby reducing the surface hardness of the material [13, 14].

Wet environment and various loading are the main conditions encountered in the mouth during mastication. Many studies have shown that wet environment significantly affected mechanical properties of dental ceramics. For example, White discovered that the strength of feldspathic porcelain exposed to moisture was lower than that in the dry environment because of the stress corrosion [15]. Similarly, Borges demonstrated that fatigues of dental ceramic crowns with fracture loading in a wet environment were statistically lower than those in a dry environment [16]. On the other hand, Salazar Marocho found that the wet environment caused pronounced subcritical crack growth in zirconia-reinforced and glass-infiltrated alumina-based ceramics [17]. The subcritical crack propagation in these materials is attributed to a combined effect of stress corrosion by water molecules at the crack tip and mechanical degradation of the material under cyclic loading. According to Studart’s study, dental ceramics containing zirconia were more prone to cyclic fatigue than lithium silicate glass–ceramic in wet environment [18]. Therefore, it is evident that the mechanical properties of dental ceramics are different in wet and dry environments according to the current literature.

At light loads of less than 10 KG, water was proved to act as a lubricant to reduce the wear rate of enamel while dry environment resulted in more enamel wear [19]. In the engineering field, during the wear test of Si_3_N_4_–hBN/Si_3_N_4_ pairs sliding with full-immersion water lubrication, hydrodynamic lubrication was obtained, which reduced the friction coefficient to 0.01 [20]. Therefore, it would be interesting to determine the effect of wet environment on the wear resistance of glass–ceramics.

The present work examined wear performance of two types of dental monolithic glass–ceramics (lithium disilicate glass–ceramics and leucite-based glass–ceramics) under water and dry conditions to simulate a serous oral environment and an extreme xerostomic oral environment, respectively. The objective of this study is to evaluate the effect of an aqueous environment on wear resistance of typical dental glass–ceramics. The hypothesis of this study is the wet environment does not affect the wear resistance of these two glass–ceramics systems.

## Methods

### Glass–ceramics and steatite specimen preparation

Two types of commercial glass–ceramics were used in this study (Table 1). Organic glass disks with the dimension of 12 mm × 3 mm (diameter × thickness) were made to replace the wax patterns. Then two pressable glass–ceramics (IPS Empress Esthetic and IPS e.max Press) were respectively fabricated into disk specimens (12 mm in diameter, 3 mm in thickness, N = 8/material) according to the manufacturing instruction. The lateral faces of the glass–ceramics specimens were subsequently polished with a rotational polishing device using 600-, 1000-, 1500-, 2000-, 2500- and 3000-grit silica carbide abrasive papers under a steady stream of water. Final dimension was 12 × 2.5 mm^3^ (diameter × thickness). One specimen of each material was randomly selected for X-ray diffraction analysis (XRD). Disk specimens of each material were embedded and fixed in the middle of a round stainless-steel mold using self-curing resin to fit the jig of the wear test device. The steatite antagonists were directly shaped into a cylinder with a diameter of 2.5 mm, which was connected to a hemisphere with a radius of 3 mm.

**Table 1:** Tested ceramic systems

Type	Code	Material	Manufacturer	Technique
Lithium disilicate glass–ceramic	LD	IPS e.max Press	Ivoclar Vivadent, Schaan, Liechtenstein	Pressing
Leucite reinforced glass–ceramic	LEU	IPS Empress Esthetic	Ivoclar Vivadent, Schaan, Liechtenstein	Pressing

### Wear testing

To simulate the occlusal contact wear, the disk specimens were aged in a two-body pin-on-disk wear testing machine (CSM Instruments, CH-2034 Peseux, Switzerland), where vertical loading was applied during cyclic loading with a constant occlusal load of 10 N. Glass–ceramic disk specimens were mounted in the lower stations running in a circular motion at a rotation speed of 200 rpm against the fixed steatite antagonists in the upper stations for 500,000 cycles. Wear tests for each material were conducted separately in distilled water and dry environments. For investigating the processive wear behavior of the glass–ceramics in different environments, the specimens per material and wear environment were subjected to 1.0, 3.0 and 5.0 × 10^5^ chewing cycles.

### Wear quantification

Volume loss was used to determine the quantitative wear data of glass–ceramic disk specimens. After 100,000, 300,000 and 500,000 chewing cycles, the substance loss on the specimen surfaces was measured using a non-contact 3D white light profilometer (PS50, Nanovea, Irvine, CA, USA) and its dedicated software (Nanovea 3D Software, Nanovea, Irvine, CA, USA). The scanned area was 7 mm × 7 mm that covered the entire wear surface. All scans were obtained at a 100 Hz frequency using a step size of 20 μm in both the x and y directions. As the wear test proceeded, the volume loss of ceramics at all checkpoints was evaluated in sequence. For steatite antagonists, volume loss was calculated by measuring the weight and density using high accuracy balance and Archimedes’ method at each checkpoint.

### Secondary electron imaging (SEM)

After the wear tests, the worn surfaces were ultrasonically cleaned. Glass–ceramic disk specimens at the last checkpoint were sputter coated (MC 1000, Hitachi High-Technologies Corporation, Tokyo, Japan). The detailed microstructural characterizations of the as-worn and cleaned surfaces were performed using a scanning electron microscope (S-4800, Hitachi High-Technologies Corporation, Tokyo, Japan).

### X-ray diffraction analysis (XRD)

Since the wear tests were performed in dry and wet environments, it is necessary to study the influence of water environmental conditions on the microstructure of glass–ceramics. One specimen of each material was cleaned ultrasonically with acetone for 30 s. The crystal phase composition of the glass–ceramic specimens was measured using a X’Pert Pro diffractometer with Cu-*K*_α_ radiation (λ = 0.15418 nm). The diffraction peaks were measured in the 2θ range from 10° to 80° with a scan step of 0.033°. Subsequently, they were immersed in the tube with distilled water for 40 h that corresponded to the period of the wear test in a wet environment. After specimens were removed and cleaned ultrasonically with acetone for 30 s, XRD analysis was conducted again. The effect of water on the crystal composition of ceramics was compared and analyzed.

### X-ray photoelectron spectroscopy analysis (XPS)

Chemical bonding states of the native surface, as well as the worn surface produced in dry and wet environments, were investigated by X-ray photoelectron spectroscopy (XPS) (AXISULTRA, Kratos, UK). XPS spectra were obtained with monochromatic Al-K_α_ (1486.71 eV) line at a power of 100 W (10 mA, 10 kV) under the vacuum about 10^–8^ Torr. The charge neutralizer was used to compensate for surface charge effects, and binding energies were calibrated using the C 1*s* hydrocarbon peak at 284.8 eV.

### Statistical analysis

Descriptive statistics, such as means and standard deviations, were computed for the material wear. The distributions of the measurements for material wear were examined using normal probability plots and the Shapiro–Wilk test, and it was determined that the measurements were approximately normally distributed (IBM SPSS Statistics for Windows, Version 22.0. Armonk, NY: IBM Corp). All of the wear loss data were analyzed using analysis of variance (ANOVA) to determine whether the differences between the mean wear-loss values of the glass–ceramics and antagonists observed at the same checkpoint were statistically significant (α = 0.05). Multiple pairwise comparisons of means were performed by Tukey’s post-hoc test using GraphPad Prism version 5 software (GraphPad Software, San Diego California USA).

## Results

### Wear of specimens

Table 2 shows the material volume loss in different environments. The results of the ANOVA indicated that the wet environment significantly affected the glass–ceramics (*p* < 0.05). Post hoc tests indicated that LEU in a wet environment exhibited significantly less wear volume loss than that in dry environment at each checkpoint. In contrast, the volume loss of LD in the wet environment was higher than that in the dry environment, which is in contrast with the results of LEU group in water and dry environments. In a wet environment, no significant differences were detected between the wear volumes of LD and LEU (*p* > 0.05). On the other hand, LD exhibited significantly lower values than LEU (*p* < 0.05) in a dry environment.

**Table 2:** Mean volume losses and standard deviations of the tested ceramics at each checkpoint in mm^3^

Groups	Chewing cycles (× 10^4^)		
	10	30	50
LD-wet	1.34 ± 0.07 A	6.38 ± 0.85 A	13.01 ± 0.69 A
LD-dry	0.36 ± 0.07 B	1.37 ± 0.09 B	2.69 ± 0.42 B
LEU-wet	1.62 ± 0.14 A	6.53 ± 0.47 A	11.84 ± 0.94 A
LEU-dry	3.64 ± 0.69 C	15.21 ± 0.81 C	34.47 ± 2.34 C

Means with the same capital letter within one row are not statistically different at *p* = 0.05 (Tukey test)

Wear volumes of steatite antagonists (Table 3) also showed statistically significant differences among the various groups. The wear volumes of steatite antagonists paired with both LD and LEU were lower under wet conditions than under dry conditions. Under wet conditions, LD caused less volume loss of antagonist than LEU (*p* < 0.05). Meanwhile, in a dry environment, the wear volume value of antagonist was also lower in the LD group than in the LEU group (*p* < 0.05).

**Table 3:** Mean volume losses and standard deviations of the tested steatite antagonists at each checkpoint in mm^3^

Groups	Chewing cycles (× 10^4^)		
	10	30	50
Antagonist with LD-wet	0 ± 0 A	0.03 ± 0.01 A	0.11 ± 0.01 A
Antagonist with LD-dry	0.1 ± 0.03 B	0.37 ± 0.04 B	0.79 ± 0.09 B
Antagonist with LEU-wet	0.48 ± 0.08 C	1.93 ± 0.23 C	4.01 ± 0.07 C
Antagonist with LEU-dry	0.64 ± 0.06 D	3.06 ± 0.25 D	7.09 ± 0.49 D

Means with the same capital letter within one row are not statistically different at *p* = 0.05 (Tukey test)

### SEM analysis

**Fig. 1:**
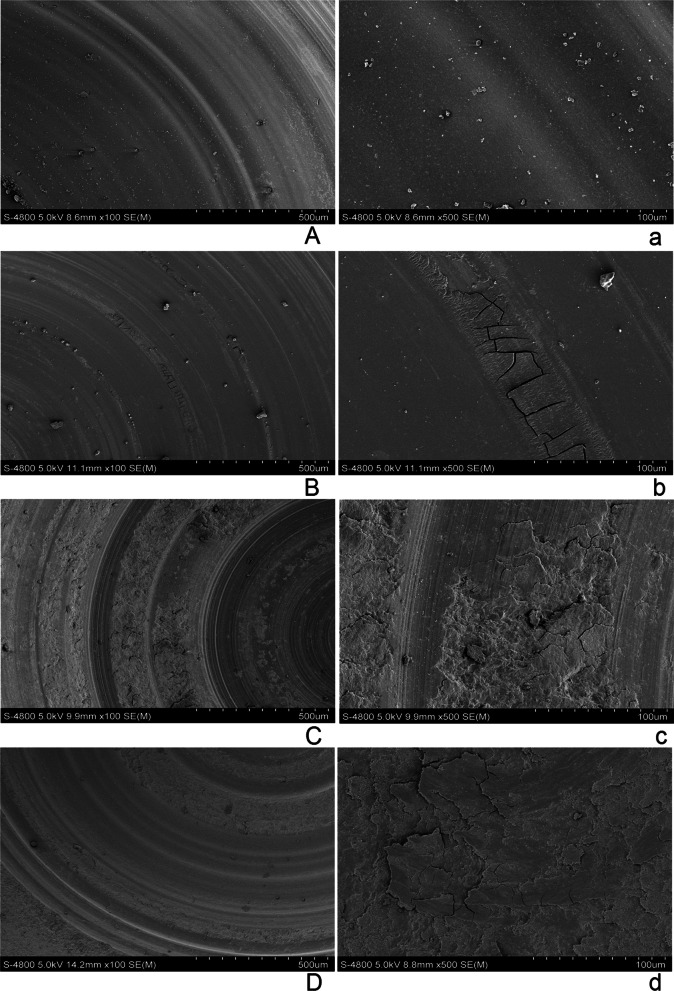
SEM images of the wear morphology of two glass–ceramics in wet and dry environments: lithium disilicate glass–ceramics in a wet environment (**A** and **a**) and in a dry environment (**B** and **b**); leucite reinforced glass–ceramics in a wet environment (**C** and **c**) and in a dry environment (**D** and **d**)

SEM images of the wear surfaces of the tested ceramic specimens in wet and dry environments under 100× and 500× magnification are presented in Fig. 1. The surface material loss of LD in a wet environment was homogenous, and the track area revealed single continuous grooves without cracks (Fig. 1A and a). In a dry environment, the relatively smooth surface of LD had superficial furrows and small local cracks (Fig. 1B and b). In a wet environment, LEU showed superficial grooves and local delamination (Fig. 1C and c). In a dry environment, the surface of LEU displayed deep furrows and interwoven cracks which resulted from the delamination and fragments stripping (Fig. 1D and d).

**Fig. 2:**
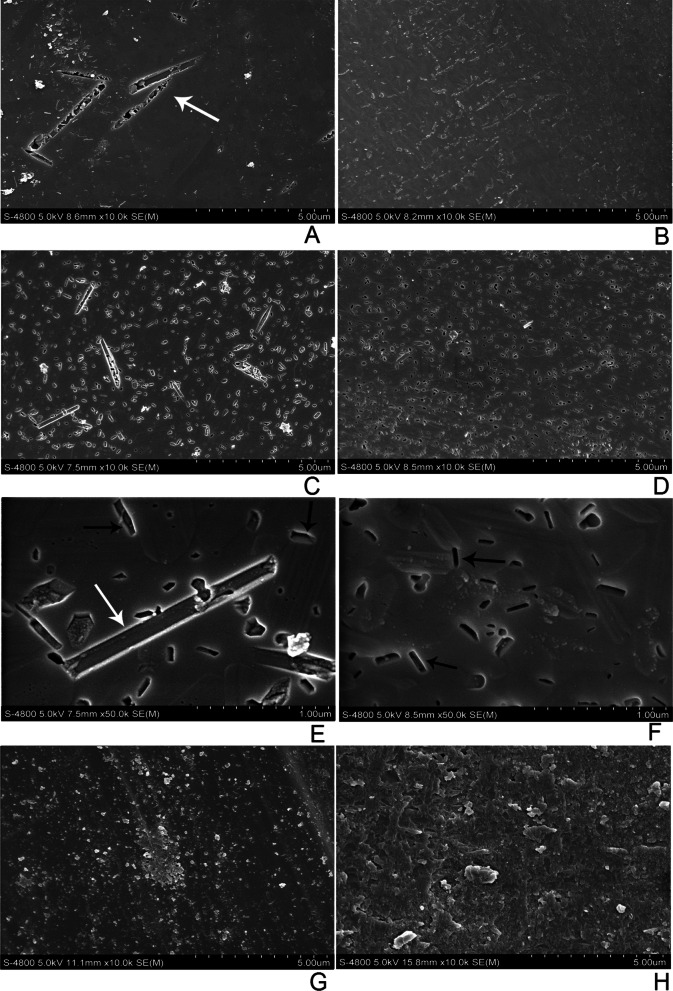
SEM images under high magnification of the as-worn surfaces developed in different environmental conditions: LD in a wet environment without ultrasonic cleaning (**A**) and after removal of the tribofilm with ultrasonic cleaning as revealed in **C** and **E**; LD in a dry environment without ultrasonic cleaning (**B**) and after ultrasonic cleaning (**D**) and (**F**); LEU in a wet environment (**G**); LEU in a dry environment (**H**); the white arrow indicates needle-like hole; the black arrow indicates the small micropores along the edge of LD crystals

The surface morphology of LD in the wet environment was quite different than that in the dry environment under high magnification. In wet conditions, needle-like holes similar to lithium silicate crystals in size and shape could be observed on the surface of LD (see Fig. 2A white arrow). After ultrasonic cleaning, the surface of LD displayed dense micropores and needle-like holes that were also more distinct as revealed in Fig. 2C and E. These microspores and holes could be generated after lithium silicate crystals were removed in the wear process. It was also observed that small pores distributed along the edge of lithium disilicate crystals. In contrast, before ultrasonic cleaning, no small pores were observed all over the wear pit, which may indicate that the wear surfaces in wet and dry conditions were covered by tribofilm (Fig. 2A and B). The chemical property of the tribofilm was further analyzed by XPS and discussed in the following section. For LEU, there were not small pores and tribofilm on the wear surface, which is different from LD. The surface of LEU in the dry environment was rough with dense granular particles. Conversely, the wear surface of LEU in wet environment displayed small particles.

### XRD analysis

**Fig. 3:**
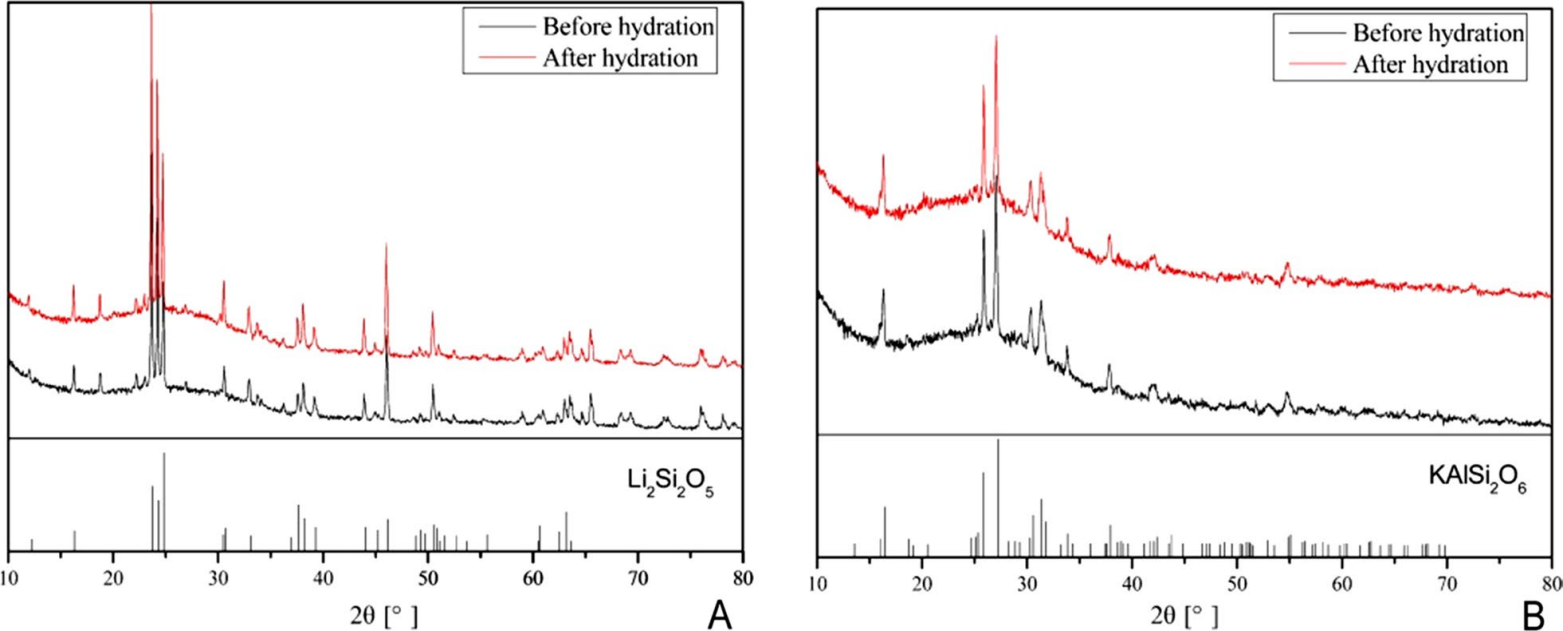
XRD patterns of two glass–ceramics in different environmental conditions: **A** LD; **B** LEU

Figure 3 shows the XRD patterns of the two types of glass–ceramics before and after hydration over a 2θ-range from 10° to 80° with a 0.033° step interval. The diffraction peaks with relatively strong intensities in LD could be indexed as lithium disilicate phase (Li_2_Si_2_O_5_), which was the main crystalline phase whereas LEU was identified as tetragonal leucite (KAlSi_2_O_6_). According to the XRD data, the width and intensity of crystal diffraction peaks of LD and LEU did not change at all before and after hydration. Therefore, it was concluded that water did not affect the crystalline phases of LD and LEU.

### XPS analysis

**Fig. 4:**
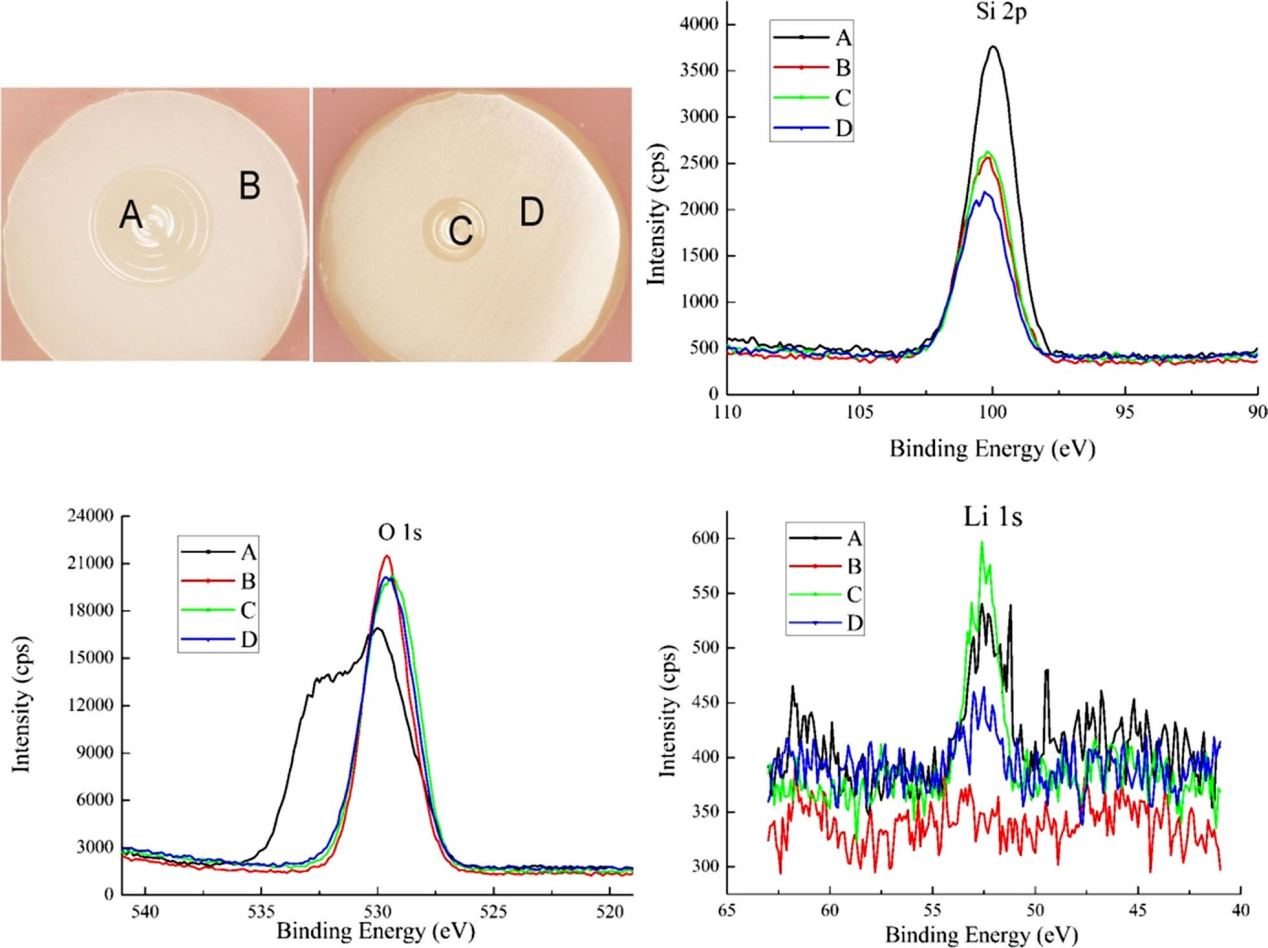
Si 2*p*, O 1*s* and Li 1*s* XPS spectra of LD specimens: **A** wear area in a wet environment; **B** outside wear area in a wet environment; **C** wear area in a dry environment; **D** outside wear area in a dry environment

The XPS experiments were carried out to analyze the composition and the chemical changes of the worn surfaces of LD produced in different conditions. The measurements were performed in and outside the wear area of LD. The results of the measurements are shown in Fig. 4. The raw XPS spectra collected from both the native surface and the wear surface over a wide binding energy (BE) region clearly showed characteristic peaks of lithium, silicon, and oxygen. The surface composition and calibrated binding energies of the major elements present in different worn surfaces are shown in Table 4.

**Table 4:** Surface composition and binding energies (eV) of LD in wet and dry conditions

LD	Li 1*s*	Si 2*p*	O 1*s*	P 2*p*	Na 1*s*
Wear area (in wet environment)	55.1	102.5	532.5	133.6	1072
Outside wear area (in wet environment)	57.1	102.9	532.3	134.1	1072.1
Wear area (in dry environment)	55.3	102.9	532.1	133.5	1072.3
Outside wear area (in dry environment)	54.9	102.7	532	133.2	1072.1

## Discussion

The lubrication effect of water on wear performance of materials is a well-known phenomenon and has been investigated extensively in previous studies [19, 21, 22]. The lubricating function of water is attributed to the removal of wear debris and hydrostatic lifting [21]. It is notable that our results slightly differ from a previous report that water played a positive role in reducing wear of materials while the dry environment resulted in more wear [22]. The present work demonstrated that water not only played a dominant role in lubrication of LEU but also resulted in aggravated wear of LD. Results of wear testing in this work showed that wet environment affected wear resistance of glass–ceramics significantly. Therefore, the original hypothesis of this study is invalidated.

The microstructure of LD consists of a glass matrix and needle-like interlocking lithium disilicate crystals (approx. 62.74%), Li_2_Si_2_O_5_. According to the microstructure of LD, the strength and fracture toughness of LD are usually much higher than those of LEU [23]. Under dry conditions, abrasive particles exist between LD and antagonist. The microscopic plowing effect caused by the wear debris becomes the dominant wear mechanism of materials. Since LD has high fracture toughness and high strength, it has a high capacity to resist microscopic plowing effect from wear debris and shows high wear resistance. As a result, the wear loss of LD was lower than that of LEU under dry conditions, as shown in Table 2. The results indicated that the wear volume loss of LD was five times higher under wet conditions than under dry conditions at 100,000, 300,000 and 500,000 chewing cycles, which is in stark contrast with the result of LEU. As shown in Fig. 2C and E, needle-like holes (white arrow) were similar to lithium silicate crystals in size on the abrasive surface of LD under wet conditions, which may be generated after the removal of lithium silicate crystals in the wear process. However, crystal morphology was evident on the wear surface of the dry environment (Fig. 2D and F) without pulling out grains. It is well known that there exists microscopic residual stress inside LD caused by the mismatch between the thermal expansion coefficients (TEC) of the crystal phase and the corresponding glass matrix in the process of cooling after controlled crystallization [24]. The average linear TECs of the orthorhombic lithium disilicate crystal phase and the corresponding glass matrix were estimated to be 10.1–10.8 × 10^–6^/K and 12.2–12.8 × 10^–6^/K, respectively [24–26]. Residual compressive stresses inside the crystals along the radial direction and the residual tensile stresses in the glass matrix along the tangential direction would increase at room temperature. We speculated that micro cracks were prone to appear between crystal and glass phase because of the TEC mismatch [27]. It was likely that the above-mentioned micro cracks were characterized as the small micropores along the edge of crystals (seen in Fig. 2E and F black arrow). These defects may be enlarged by water-assisted subcritical crack growth during wear testing via a hydraulic pumping mechanism [28], resulting in the possible falling of lithium disilicate crystal. Previous studies have demonstrated that the effect of stress corrosion by water molecules from the environment and reaction of water molecules with the ionic-covalent bonds at the crack tip induced crack propagation [29], which was also confirmed by the subsequent XPS analysis. Salazar Marocho also reported that a wet environment combined with cyclic loading induced crack propagation at stress levels more than 50% below the initial strength of the material, confirming the strong susceptibility of dental ceramics to subcritical crack growth [17]. Therefore, although water could remove wear debris and generate a microscopic plowing effect on ceramics, lithium disilicate crystals are prone to fall off because of stress corrosion by water molecules. As a result, the wear resistance of LD is poorer in water environment than in the dry environment. Figure 1 also shows that wear track of LD exhibited an apparent plowing effect in water environment compared with that in the absence of water, which is consistent with the results of the wear loss.

In contrast, upon cooling after controlled crystallization, a cubic to tetragonal transformation with volume contraction occurred for the leucite phase. The TEC of the tetragonal leucite phase was much higher than that of the corresponding glass matrix [30, 31]. These two factors could cause residual tensile stresses inside the crystals along the radial direction at room temperature, which would be balanced by residual compressive stresses in the glass matrix along the tangential direction. It appears that the micro residual stress state in LEU is just opposite to that in LD, which could be the possible reason that there were no micro cracks or crystals falling off on the abrasive surface of LEU, as shown in Fig. 2G and H. The wear volume loss of LEU at each checkpoint was higher in the dry environment than in a wet environment. In the dry environment, sliding wear occurred on the interface of glass–ceramics and antagonists, where compressive stress, tensile stress, and shear stress took place repeatedly. In addition, mechanical properties such as elastic modulus, fracture toughness, and flexure strength of LEU were low [23, 32, 33]. These factors led to the formation and accumulation of debris in the contact area, thus increasing local stress and aggravating wear in a dry environment. Nevertheless, the wear debris generated in the wear process was removed from the contact area in a circular motion under wet conditions.^21^.

The worn surface and native surface of LD in dry and wet conditions were analyzed by XPS. As shown in Table 4, the binding energies obtained for the Si 2*p* core level were in the range of 102.5–102.9 eV and very close for all samples, suggesting that in each sample the bonding states of the silicon were very similar. The binding energy peak could correspond to silicon in SiO_2_ (about 103.0 eV) and silicates (102.6 eV). Indeed, LD mainly consisted of lithium disilicate crystals and glass matrix [26, 34], which was consistent with the XPS spectra. The binding energy peak of the O 1*s* obtained from the wear surface (denoted as A in Fig. 4) in the wet environment was at 532.5 eV, which could be assigned to oxygen in SiO_2_ (about 532.5 eV). The shoulder of the main peak corresponded to oxygen in bound H_2_O (532.9 eV). The main peak of the O 1*s* from the native surface (denoted as B in Fig. 4) in the wet environment was shifted to 532.3 eV without a shoulder. The chemical state of oxygen on the worn surface (A) was different than that on the native surface (B). The shape of O 1*s* recorded at position C was similar to D, which indicated that the chemical state of oxygen obtained from the worn surface (C in Fig. 4) in the dry environment was similar to that on the native surface (D in Fig. 4). The binding energy of Li 1*s* obtained from the wear surface (denoted as A) in the wet environment was at 55.1 eV, which can be assigned to Li in a Li–OH state (about 54.9 eV). Taking into account the change in the O 1*s* spectra of the wear track that indicates the existence of a high amount of bound H_2_O in the wet environment, we can assume that hydroxylated Li species are formed because of the wear process in a wet environment. In the meantime, the effect of stress corrosion by water molecules from the wet environment and reaction of water molecules with the ionic-covalent bonds at the crack tip caused aggravate wear. The Li 1*s* peak from the wear surface (denoted as C) in a dry environment was shifted to 55.3 eV, which may be assigned to Li in a Li–CO_3_ state (about 55.3 eV). Therefore, it is the change of Li chemical state that generates a tribofilm (seen in Fig. 2B) on the worn surface of LD in the dry environment, which causes less wear loss of LD than that in the wet environment (Table 2).

It is notable that steatite antagonist wear losses of the LD groups in two environments were lower than that of LEU after 500,000 cycles (*p* < 0.05), as shown in Table 3. Because of high mechanical properties of LD and the presence of tribofilm, less abrasive particles are generated in the contact area. Since LEU has relatively poor mechanical properties such as low flexure strength and fracture toughness, more abrasive particles are formed and accumulated. More abrasive particles result in increased wear loss gap between two groups with increasing chewing cycles. It is also noteworthy that steatite antagonist quantitative wear of the LD and LEU groups in the wet environment is lower (*p* < 0.05) than that in the dry environment after 500,000 cycles (Table 3). Apparently, more abrasive particles result in increased wear loss gap between two environments with increasing chewing cycles.

XRD pattern showed that the microstructures of two types of glass–ceramics did not change after immersed in water for 40 h. This illustrated that the change of wear resistance with environment might be caused by different wear mechanisms in different environments rather than by the change of crystal type and crystal volume fraction.

In this study, distilled water was used to replace saliva to simulate the oral humid environment. Due to the lack of organic substances in saliva, distilled water could not completely simulate the wear of prosthesis in the mouth. Due to the complicated oral environment and various loading conditions, there is a gap between the present results and real wear. Nevertheless, the results of this work may serve as a valuable reference for reasonable selection of dental ceramic restorations, thus helping to achieve the ultimate purpose of low wear and low damage. For example, LD is a better choice for patients with xerostomia compared with LEU.

## Conclusions

Within the limitations of the present study, the following conclusions were drawn:

1. A wet environment causes more wear loss of LD paired with steatite than a dry environment.
2. Dry friction produces more wear loss for LEU, whereas a wet environment plays a lubricating role.
3. The XRD results revealed simple wet environment hardly influenced crystalline phases of LD and LEU.
4. XPS spectra from the worn surface of LD under wet conditions showed the existence of a high amount of bound H_2_O, which might imply that high wear loss of LD in the wet environment results from the effect of stress corrosion by water and reaction of water with the ionic-covalent bonds at the crack tip. XPS spectra from the worn surface of LD under dry conditions suggested that the possible tribofilm formation of Li–CO_3_ state, which may serve as a protective lubricating layer in reducing the friction and wear of LD when it is against steatite under dry conditions.

## Data Availability

All data generated or analysed during this study are included in this published article.
